# Antiretroviral Therapy with Ritonavir-Boosted Atazanavir- and Lopinavir-Containing Regimens Correlates with Diminished HIV-1 Neutralization

**DOI:** 10.3390/vaccines12101176

**Published:** 2024-10-17

**Authors:** Eloisa Yuste, Horacio Gil, Felipe Garcia, Victor Sanchez-Merino

**Affiliations:** 1 National Microbiology Center, Institute of Health Carlos III (ISCIII), 28220 Madrid, Spain; hgil@isciii.es (H.G.); vmsanchez@isciii.es (V.S.-M.); 2 Centro de Investigación Biomédica en Red de Enfermedades Infecciosas (CIBERINFEC), 28029 Madrid, Spain; 3 Infectious Diseases Department, Hospital Clínic, University of Barcelona, 08036 Barcelona, Spain; fgarcia@clinic.cat

**Keywords:** HIV-1, Broadly neutralizing antibodies, ART, undetectable viremia, boosted protease inhibitors

## Abstract

Background/Objectives: The impact of virion maturation on neutralizing antibody responses in HIV treatment is not fully understood. This study examines whether antiretroviral regimens (ART) with boosted protease inhibitors (b-PI), which increase exposure to immature virions, affect neutralization capacity compared to Non-b-PI regimens. Methods: Neutralization activity was assessed in 45 HIV-infected individuals on b-PI regimens and 56 on Non-b-PI regimens, adjusting for factors like infection duration, ART initiation, and immune markers. Individuals on b-PI regimens had significantly lower neutralization scores [mean: 6.1, 95% Confidence Interval (CI): 5.3–6.9] than those on Non-b-PI regimens (mean: 8.9, 95% CI: 8.0–9.9; *p* < 0.0001). This difference was not explained by infection duration or CD4+ counts. CD4+/CD8+ ratios were positively associated with neutralization, while b-PI use was negatively associated. A regression model indicated that b-PI use significantly predicted lower neutralization scores (beta = −0.30, *p* = 0.049). Conclusions: These findings suggest that exposure to immature virions via b-PI use reduces neutralizing antibody responses, highlighting the importance of virion maturation in antibody induction. ART regimens promoting exposure to mature virions may enhance neutralization, with potential implications for HIV vaccine design. Further research is needed to explore implications for HIV vaccine design, especially using virus-like particles.

## 1. Introduction

The lipid envelope of HIV-1 contains gp120/gp41 glycoprotein trimers responsible for binding to and fusing with host cell membranes, crucial for the virus’s infectivity. These HIV-1 Env proteins are integrated into the virus particle through interactions between the gp41 C-terminal tail and the viral structural polyprotein Gag. Studies using biochemical and cryo-electron microscopy techniques have shown that SIV and HIV-1 particles typically possess only 7 to 14 Env trimers, in contrast to other enveloped viruses, which usually have a higher density of viral entry proteins on their surfaces [1,2,3,4,5]. During budding, the hexameric Gag lattice beneath the viral membrane disassembles into its constituent domains via HIV-1 protease (PR)-mediated cleavage, leading to the formation of the mature infectious HIV-1 structure characterized by its cone-shaped capsid [6]. However, immature or partially matured HIV-1 particles exhibit reduced entry efficiency that reverts upon truncation of the gp41 cytoplasmic domain [7,8,9,10,11]. This diminished efficiency requires interactions between the Gag protein and the Env cytoplasmic domain. It may be due to the rigidity of the immature Gag lattice, which restricts membrane fusion, or to changes in Env lateral mobility that hinder the clustering of sparsely distributed Env trimers.

The movement of spikes on the virion surface and spike cluster formation have been characterized thanks to stimulated emission depletion (STED) and super-resolution fluorescence microscopy (SRFM) studies. These studies revealed that the surface of immature HIV-1 particles is characterized by multiple separated Env molecules, whereas the surface of mature particles features only a single Env cluster [12,13]. Interestingly, the effect of cluster formation on the antigenicity of the spikes is currently unknown. This emerging understanding of Env clustering dynamics is particularly relevant in the context of broadly neutralizing antibodies (bNAbs), which may depend on interactions between Env spikes.

For certain broadly neutralizing antibodies, recognition has been shown to depend on inter-spike interactions, which could be influenced by cluster formation [14,15,16]. Consequently, due to these dynamics, the same HIV spike may vary in its ability to induce bNAbs depending on whether it is presented in mature or immature virions. Indeed, it has been reported that the expression of specific gp41 epitopes differs between mature and immature virions [11]. Therefore, characterizing how cluster formation affects bNAb epitope conformation is crucial for designing vaccine prototypes based on virus-like particles that replicate the structure of HIV-1 virions.

HIV-1 protease, as previously mentioned, acts during the budding of new virions from the host cell by cleaving viral polyprotein precursors (Gag and Gag-Pol) produced by the infected cell into individual structural proteins and enzymes. This cleavage process converts morphologically immature virions into mature, infectious particles [17]. In HIV-1 treatment, protease inhibitors (PIs) have become one of the most important components of combination therapy due to their ability to inhibit viral protease enzymatic activity. Consequently, without proper proteolytic cleavage, virions remain immature and non-infectious. To understand these effects comprehensively, it is also crucial to consider how antiretroviral therapy (ART) influences the maturation of HIV-1 particles and the subsequent clustering of Env proteins.

Currently, ten HIV protease inhibitors (PIs) are available: saquinavir, indinavir, ritonavir, nelfinavir, amprenavir, fosamprenavir, lopinavir, atazanavir, tipranavir, and darunavir. These drugs are predominantly metabolized in the liver, where they undergo biotransformation via the cytochrome P450 (CYP) enzyme system, particularly the CYP3A4 isoenzyme. PIs are often administered with a low dose of ritonavir or cobicistat, which serve as pharmacokinetic boosters. Ritonavir, though itself a PI, is used in subtherapeutic doses to inhibit CYP3A4, thereby slowing the breakdown of the primary PI and maintaining higher, more sustained plasma levels. Similarly, cobicistat, while not an antiretroviral agent, inhibits CYP3A4 to enhance the PI’s effectiveness [18]. This boosting strategy allows for less frequent dosing, improves patient adherence, and increases antiviral activity by ensuring higher and more consistent drug concentrations. Additionally, boosted PIs are effective in managing drug-resistant HIV strains and may offer a more favorable safety profile due to reduced dosing requirements [19,20]. Among these boosted-PI regimens are atazanavir/ritonavir and lopinavir/ritonavir.

Given that ART regimens with and without protease inhibitors can affect the maturation of HIV-1 particles and, consequently, the distribution of Env proteins, investigating the impact of these treatments on the immunogenicity of HIV-1 spikes is essential.

A scenario in which the differences in the immunogenicity of HIV spikes, due to their formulation in mature or immature virions, could be investigated is in HIV-1 infected individuals on ART regimens with and without PIs. The rationale behind this approach is that, in ART regimens including PIs, the majority of antigenic stimulation occurs with HIV-1 spikes presented on the surface of immature virions. In contrast, in ART regimens without PIs, a significant portion of antigenic stimulation occurs in the presence of mature HIV-1 spikes presented in mature virions. The main difficulty with this approach is that the neutralizing response against HIV is significantly reduced in the presence of an effective ART regimen. However, in previous studies, we have shown that, although reduced, broadly neutralizing responses can be detected in the absence of viremia [21,22].

In the present study, we investigated the ability to induce broadly neutralizing responses through antigenic stimulation with virions of mature and immature morphology. To this end, we used data from a previous study that characterized anti-HIV antibody-mediated neutralization in a group of individuals with ART-suppressed viremia and compared the neutralizing responses induced in the presence or absence of antiretroviral therapy with ritonavir-boosted atazanavir-and-lopinavir-containing regimens.

## 2. Materials and Methods

### 2.1. Study Participants

**Figure 1:**
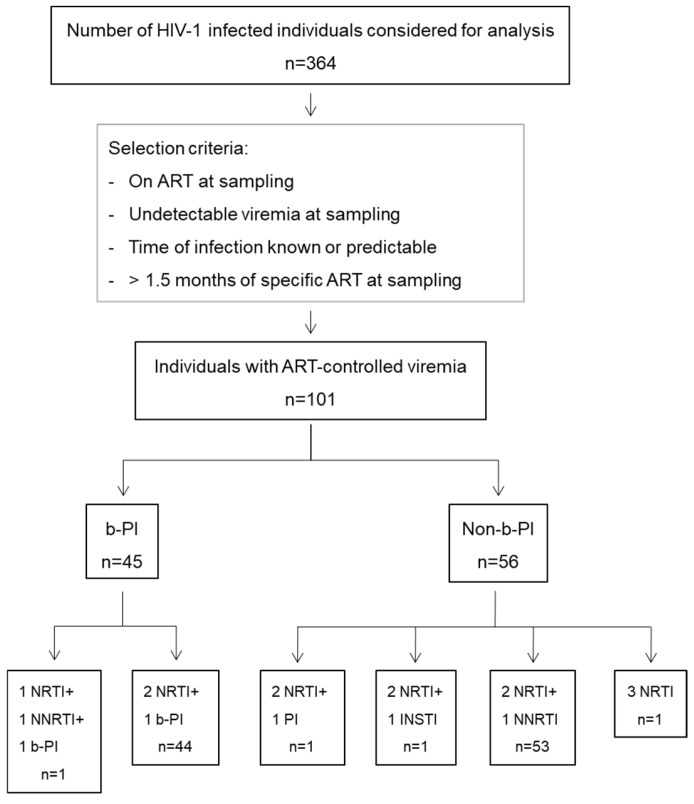
Individual selection and stratification. Serum-purified IgGs from 364 individuals were included. b-PI: ART regimen including boosted protease inhibitor (protease inhibitor + ritonavir). Non-b-PI: ART regimen not including boosted protease inhibitor. NRTI: nucleoside analog reverse-transcriptase inhibitor; NNRTI: non-nucleoside analog reverse-transcriptase inhibitor; PI: protease inhibitor; and INSTI: integrase strand transfer inhibitor.

The neutralization data used in this study were obtained from a previous study including samples from 364 people living with HIV-1 (PLWH) treated at the Hospital Clinic of Barcelona, Spain. For the present study, we selected neutralization data from 108 individuals who had suppressed viremia due to ART at the time of sampling [21]. Given that the purpose of this study is to determine the effect of specific antiretroviral regimens on neutralization, we excluded 7 individuals who had been on a specific treatment regimen for less than 1.5 months at the time of sampling. Of the 101 study participants, 45 (44.6%) were on an ART regimen that included a b-PI, while 56 (55.4%) were on a regimen without b-PI. Among those on the b-PI regimen, the majority of individuals (44 (43.6%)) were on a combination of two nucleoside reverse-transcriptase inhibitors (NRTIs) and one b-PI, and 53 (52.4%) were on a regimen of two NRTIs and one non-nucleoside reverse-transcriptase inhibitor (NNRTI) in the group on a regimen without b-PI (Figure 1 and Table 1). The median age of the participants was 42 years, with a median time since infection of 8.7 years and a median duration on ART of 1.3 years. All individuals had a suppressed viral load (<50 RNA copies/mL) and a median CD4+ count of 660 cells/mm^3^, with a CD4+/CD8+ ratio of 0.7. The complete demographic, ART regimen, and clinical characteristic data of the study participants are shown in Table 1.

### 2.2. Neutralization Assays

Neutralization assays have been described previously [21,22]. Briefly, IgGs purified from heat-inactivated plasma were tested in triplicate at 0.2 µg/mL with a virus minipanel consisting of six recombinant replication-competent viruses from five different genetic subtypes. As specificity control, an amphotropic vesicular stomatitis virus Env pseudotype was added to the panel. A cumulative score was calculated for each plasma sample, considering the neutralization percentages against all six viruses. This was carried out using a previously established scoring system based on the percentage of inhibition of each virus from the panel, using a single plasma IgG concentration (0.2 µg/mL) [22].

### 2.3. Statistical Analysis

The comparison of the qualitative variables with the neutralization scores was performed using Mann–Whitney U and Kruskal–Wallis tests. The association between the quantitative variables and the neutralization scores was analyzed using Spearman correlation analysis. A multivariable analysis using a multiple linear regression model was performed to identify predictors of the neutralization response in study participants. Standardized beta coefficients were calculated to compare the effect of each predictor variable in the model. *p*-values < 0.05 were considered statistically significant. Data analyses were performed using the STATA statistical software package Version 18 (Stata Corporation, College Station, TX, USA).

## 3. Results

### 3.1. Neutralizing Antibody Response in Aviremic HIV-1 Infected Individuals Treated With vs. Without Boosted Protease Inhibitors

To determine the effect of virion maturation on induced neutralizing responses in our cohort, we compared the neutralization scores of individuals on ART that included a b-PI, which provides antigenic stimulation primarily with immature virions, with those on Non-b-PI ART, which provides antigenic stimulation with mature virions. The b-PI group was chosen as the one exposed to immature virions because previous reports indicate that efficient protease inhibition, and thus, exposure to immature virions, is achieved in the presence of ritonavir [23]. Within our cohort, there is one individual whose treatment regimen included an unboosted protease inhibitor (without ritonavir) for 16 months. This individual was included in the group primarily exposed to mature virions because, according to previous reports [23], unboosted-PI treatment does not ensure efficient inhibition of the viral protease and, consequently, we considered that, under these conditions, the exposure to virions with immature morphology had not been sufficient.

**Figure 2:**
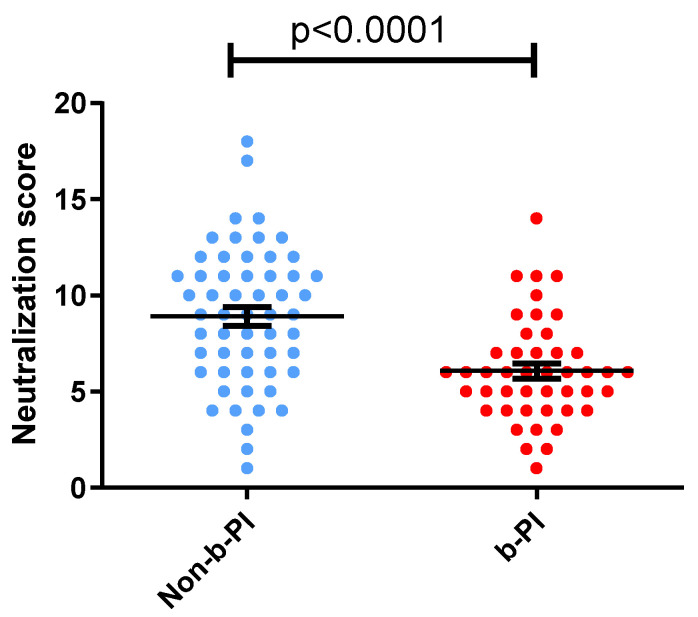
Comparison of neutralizing antibody responses (neutralization scores) in individuals treated with and without boosted protease inhibitors. HIV-1 infected individuals were on treatment regimens that either included boosted protease inhibitors (b-PI) or did not (Non-b-PI). Horizontal bars in the box plots indicate the mean for each group, and the standard errors of the means (SEMs) are shown.

In our cohort of 101 participants, the mean neutralization score was 7.6 [95% confidence interval (CI): 7.0–8.3]. In the b-PI ART group (45 individuals), the mean neutralization score was 6.1 (95% CI: 5.3–6.9). However, the mean neutralization score in individuals on the Non-b-PI ART regimen (56 individuals) was 8.9 (95% CI: 8.0–9.9) (Figure 2). When comparing both cohorts, we observed significant differences in neutralization scores (*p* < 0.0001) (Figure 2). Detectable neutralization breadth (cross neutralization-scores 5–9; broad neutralization-scores 10–13; and elite neutralization-scores 14–18) was observed in samples from 49 out of 56 Non-b-PI individuals (87.5%). However, only 33 (1 elite, 4 broad, and 28 cross-neutralization) out of 45 b-PI individuals (73.3%) showed detectable neutralization breadth (Table 2). Next, we analyzed other factors that could have contributed to the differences in neutralization score values observed between both groups.

### 3.2. The Neutralization Differences between Individuals Treated With and Without b-PI Cannot Be Attributed to Other Factors Previously Associated with Neutralization Capacity

The induction of broadly neutralizing responses has previously been associated with factors such as long periods of infection, time to ART initiation, CD4+ T-cell counts, CD4+/CD8+ ratios, and nadir CD4+ T cell counts [22,24,25,26,27]. To ensure that the observed differences in neutralization scores were not influenced by these factors, we compared these variables between the Non-b-PI and the b-PI groups.

**Figure 3:**
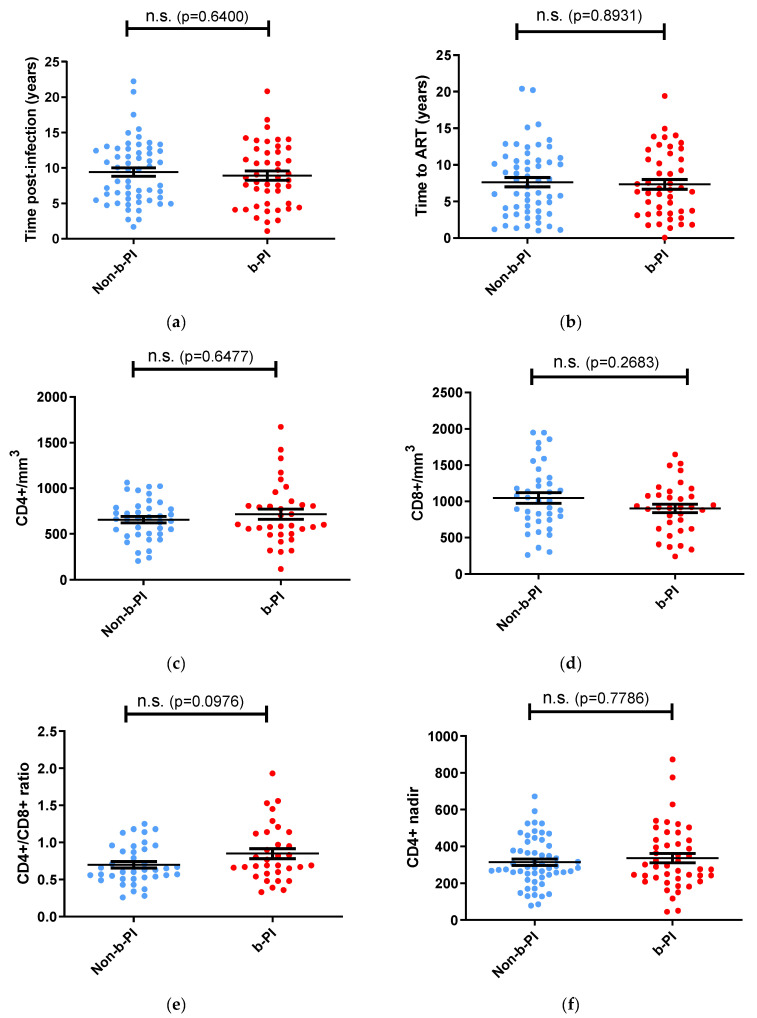
Comparison of cumulative time post-infection, time to ART initiation, CD4+ and CD8+ T-cell counts, CD4+/CD8+ T-lymphocyte ratios, and nadir CD4+ T-lymphocyte counts between individuals treated with and without b-PI. (**a**) Time post-infection comparison. (**b**) Time to ART initiation comparison. (**c**) CD4+ T-cell count comparison. (**d**) CD8+ T-cell count comparison. (**e**) CD4+/CD8+ T-lymphocyte ratio comparison. (**f**) Nadir CD4+ T-lymphocyte count comparison. Horizontal bars within the box plots indicate the mean for each group, and standard errors of means (SEMs) are shown. Significance levels between groups are indicated. Mann–Whitney U tests were used for comparisons between groups. Simple comparisons were made with two-sided alpha level of 0.05.

Our analysis reveals no statistically significant differences in the duration of infection, time to treatment initiation, CD4+/CD8+ ratios, CD4+ counts, CD8+ counts, and nadir CD4+ counts between the two groups (Figure 3). These results support the hypothesis that antigenic stimulation with mature virions promotes neutralization. To further validate this hypothesis, we conducted additional univariate analyses to identify factors associated with neutralization score values in our cohort of patients with ART-controlled viremia.

### 3.3. Univariate Analyses Confirmed the Association Between Neutralization Score Values with CD4+/CD8+ Ratios and Specific ART Regimens

Next, we used univariate analyses to identify the variables that, either singly or in combination with others, were associated with neutralization in our cohort of PLWH with ART-controlled viremia. Regarding other factors previously associated with neutralization [22], the present study confirmed that CD4+/CD8+ ratios were positively associated with neutralization. However, the time until ART initiation, despite being close (*p* = 0.07), did not reach the statistical significance threshold (Table 3).

These analyses also revealed a negative association with two different b-PI ART regimens (Table 4). The mean neutralization score for individuals treated with atazanavir + ritonavir was 6.1 (95% CI: 5.3–7.0), which was 1.3 times lower than the score for individuals whose ART did not include this drug combination. Similarly, the mean neutralization score for individuals treated with lopinavir + ritonavir was 1.4 times lower than for those treated without this combination (6.0; 95% CI: 4.6–7.4). In contrast, neutralization scores were positively associated with efavirenz-containing ART regimens, with a mean neutralization score that was 1.4 times higher compared to those of individuals on treatments not including efavirenz (mean neutralization score with efavirenz: 9.1; 95% CI: 8.1–10.0). When interpreting these results, it must be considered that the majority of individuals treated with efavirenz belonged to the Non-b-PI group (95.7%, Table 4).

### 3.4. A Multiple Linear Regression Analysis Corroborates b-PI Regimen as a Neutralization-Associated Signature

After selecting the statistically significant variables, along with time to ART initiation—which has been previously associated with neutralization scores [22]—a multiple linear regression was conducted to test whether CD4+/CD8+ ratios, the use of b-PI or efavirenz in the ART regimens, and time to ART significantly predicted the neutralization response of PLWH with controlled viremia. The overall regression was statistically significant [F(4, 67) = 6.82; *p* = 0.0001], with 25% of the changes in the neutralization score predicted by this model (Adjusted R^2^ = 0.25).

The presence of b-PI in the regimens was significant in predicting antibody neutralization responses (*p* = 0.049, Table 5), having a negative impact on neutralization score values. Moreover, b-PI contributed the most among the different predictors in the regression (beta = −0.30). According to this model, the presence of b-PI in the ART regimen decreased neutralization scores by 2.1 points (Table 5). In contrast, efavirenz (*p* = 0.269) did not significantly predict neutralization scores. Notably, the two factors that have been previously associated with neutralization-CD4+/CD8+ ratios and time to ART initiation, despite being very close, did not reach the statistical significance threshold (*p* = 0.084 and *p* = 0.081, respectively) (Table 5).

## 4. Discussion

Our study explores the impact of virion maturation on neutralizing antibody responses in individuals with ART-controlled HIV infection. We hypothesized that exposure to immature virions, influenced by the use of b-PI, would adversely affect the induction of broadly neutralizing responses compared to exposure to mature virions. Our findings reveal significant differences in neutralization scores between individuals on ART regimens including b-PI and those on regimens without b-PI, supporting our hypothesis and highlighting the critical role of virion maturation in shaping immune responses [28,29].

Our data indicate that individuals on ART regimens including b-PI (which tend to stimulate the production of immature virions) have a mean neutralization score of 6.1 (95% CI: 5.3–6.9), significantly lower than the 8.9 (95% CI: 8.0–9.9) observed in those on regimens without b-PI (*p* < 0.0001). This difference underscores the influence of virion maturation on neutralization capacity. The lower neutralization scores associated with b-PI regimens align with previous studies suggesting that the maturity of HIV-1 virions affects the development of neutralizing antibodies [11]. Immature virions, more prevalent in the presence of b-PI, might present a less effective target for neutralizing antibodies compared to mature virions. This finding supports the notion that antigenic stimulation with mature virions enhances the likelihood of developing broadly neutralizing antibodies. However, we cannot rule out that modifications in the Env protein related to b-PI treatment may also be contributing to this effect, regardless of the maturation state of the virions. In fact, the association between changes in the Env protein and PI treatment has been confirmed by the identification of PI resistance mutations located in the viral envelope gene [30]. We consider that it is important to explore this influence in future studies.

The decision to classify individuals with unboosted protease inhibitors (without ritonavir) as being exposed to mature virions is substantiated by literature indicating that unboosted PIs are less effective in achieving the virologic control by HIV protease inhibition necessary to influence neutralization responses [23]. Our approach to differentiating between b-PI and Non-b-PI groups aligns with established views on the impact of ART on HIV protease functionality and, consequently, on the maturation state of virions.

In analyzing other potential factors influencing neutralization scores, we found no statistically significant differences in the duration of infection, time to ART initiation, or CD4+/CD8+ ratios between the b-PI and Non-b-PI groups. This lack of difference reinforces the specific impact of virion maturation rather than other traditional immune parameters on neutralization responses. Previous studies have identified long-term infection, time to ART initiation, and immune cell counts as important factors in neutralization development [22,27,31,32]. However, our results suggest that these factors may not differ substantially between the two ART groups, emphasizing that the maturation state of virions is a critical determinant of neutralization efficacy.

In our univariate analyses, we confirmed the positive association between CD4+/CD8+ ratios and neutralization scores, consistent with previous studies highlighting the role of immune cell dynamics in antibody development. However, the near-significant association of time to ART initiation with neutralization, while not reaching statistical significance, suggests a potential but less pronounced role of early ART initiation in shaping neutralization responses. This finding warrants further exploration to determine if and how early ART initiation may interact with virion maturation effects.

Conversely, our analyses revealed that b-PI regimens, particularly those involving atazanavir + ritonavir and lopinavir + ritonavir, are associated with lower neutralization scores. The mean neutralization scores for these combinations were 1.3 to 1.4 times lower compared to Non-b-PI regimens, indicating a negative impact on neutralization responses. The adverse effect of b-PI on neutralization scores is particularly striking and underscores the need for careful consideration of ART regimens in terms of their impact on immune response quality.

Interestingly, efavirenz-containing regimens were associated with higher neutralization scores, though this association was not statistically significant in our regression model (*p* = 269). Indeed, efavirenz was predominantly used in the Non-b-PI group and may confound the results of the univariate analysis. In our analysis, we cannot rule out that efavirenz-containing regimens may have a favorable effect on neutralization. However, to our knowledge, treatment with this antiretroviral has not been associated with modifications in the induction of neutralizing antibodies or in the conformation of the viral spike. For this reason, we consider it more likely that the differences in neutralization observed between both groups are due to variations in the conformation of the Env protein associated with changes in the maturation state of the virions.

Our multiple linear regression analysis indicates that the presence of b-PI in ART regimens is a significant predictor of lower neutralization scores (*p* = 0.049), contributing most significantly among the predictors in our model (beta = −0.30). This result reinforces the hypothesis that b-PI negatively impacts neutralization responses. The model explained 25% of the variability in neutralization scores, highlighting that while b-PI has a substantial impact, other factors not included in our model may also play a role. In order to improve this study, it would have been interesting to analyze the effect of other PI combinations, as well as other types of antiretrovirals. Unfortunately, our cohort does not have enough individuals in these ART regimens to conduct such an analysis.

Additionally, CD4+/CD8+ ratios and time to ART initiation approached but did not reach statistical significance (*p* = 0.084 and *p* = 0.081, respectively), indicating that their roles in predicting neutralization scores are less pronounced or potentially confounded by other variables. When interpreting these results, it is crucial to account for the missing data in our dataset, particularly concerning CD4+ and CD8+ counts, which limited the number of individuals available for inclusion in the regression analysis. This limitation may have impacted the statistical significance of certain predictors, such as the CD4+/CD8+ ratio or the time to ART initiation. To confirm the role of these factors in the neutralization response, future analyses should include a larger cohort of patients with complete data.

Our findings highlight that, although our study primarily focuses on the impact of virion maturation on neutralizing antibody responses, it is worth considering that bPIs may have additional functions that could also influence neutralization outcomes. Beyond their role in altering virion maturation, bPIs might affect immune responses through mechanisms such as modulating the presentation of viral antigens or influencing the activation and function of immune cells. Additionally, PIs could influence cytokine profiles or other immune regulatory pathways, further affecting the overall neutralizing antibody response. On the other hand, additional preclinical studies are needed to determine the effect of virion maturation on the antigenicity and immunogenicity of viral spikes.

Regarding the potential applications of this study, it would be valuable to explore how these findings may influence the development of ART regimens and the design of vaccines. For example, it could be beneficial for clinicians to consider the individual’s immune response when selecting ART regimens. For patients on b-PI regimens, regular monitoring of immune markers (e.g., neutralization scores, CD4+/CD8+ ratios) could help to identify those whose immune responses might be compromised, allowing for adjustments in therapy. To optimize treatment regimens, not only to suppress viral load but also to enhance immune responses, potentially improving long-term outcomes, additional studies would be necessary. In future studies, it would be interesting to analyze longitudinal data that would provide insights into the long-term impact of b-PI regimens on the breadth of neutralization.

For VLP-based vaccines, this finding suggests that the maturation state of the VLPs used as antigens could profoundly influence the vaccine’s efficacy. VLPs that mimic immature virions might not be as effective in inducing neutralizing antibodies compared to those that represent the mature virion morphology. Therefore, vaccine design should prioritize the production of VLPs that closely resemble mature virions to optimize the induction of a strong and broad neutralizing antibody response. In addition, vaccine strategies might also explore ways to ensure they display conformational epitopes similar to mature virions, which are more likely to stimulate bnAbs. In the case of passive immunotherapy, where bnAbs are administered to individuals to prevent or treat HIV infection, it might be more effective if combined with Non-b-PI regimens. This could enhance the effectiveness of administered antibodies by providing a more suitable target for neutralization.

While the present study focuses on HIV, the principle that virion maturation influences immune responses could have broader implications for other viral infections and vaccine designs. Research could explore whether this concept applies to other viruses where virion maturation is a key aspect of the viral life cycle, such as hepatitis C or influenza, potentially informing the development of new vaccines or therapeutic approaches for these diseases.

## 5. Conclusions

The evidence presented indicates that the structural and conformational state of the HIV-1 antigens, as determined by the maturation state of the virions, is a key determinant of the neutralizing antibody response. VLP-based vaccines should therefore be designed to present mature virion-like structures to maximize the induction of bnAbs, which are essential for an effective HIV-1-preventive vaccine.

## Figures and Tables

**Table 1:** Demographics, ART regimen, and clinical characteristics.

Characteristics		Individuals
**Total Number of Individuals**		**101**
Number (%) of individuals by gender	Male	89 (88.1)
	Female	12 (11.9)
Number (%) of individuals by risk group	Heterosexual	22 (21.8)
	MSM	68 (67.3)
	IDU	4 (4.0)
	No data	7 (6.9)
Number (%) of individuals with hepatitis infection ^a,b^	No	88 (87.1)
	Yes	3 (3.0)
	No data	10 (9.9)
Number (%) of individuals by ART regimen		
b-PI ^c^		45 (44.6)
	1 NRTI+1 NNRTI+1 b-PI	1 (1.0)
	2 NRTI+1 b-PI	44 (43.6)
Non-b-PI ^d^		56 (55.4)
	2 NRTI+1 PI	1 (1.0)
	2 NRTI+1 INSTI^c^	1 (1.0)
	2 NRTI+1 NNRTI	53 (52.4)
	3 NRTI	1 (1.0)
Median (range) age (yrs) ^b^		42.0 (27.0–78.0)
Median (range) time post infection (yrs) ^b^		8.7 (1.1–22.2)
Median (range) time to ART (yrs) ^b^		6.8 (0.1–20.4)
Median (range) time on ART (yrs) ^b^		1.3 (0.1–5.7)
Median (range) nadir CD4+ cells/mm^3^		289.5 (45.0–873.0)
Median (range) CD4+ cells/mm^3 b^		660.0 (117.0–1673.0)
Median (range) CD8+ cells/mm^3 b^		917.0 (243.0–1950.0)
Median (range) CD4+/CD8+ ratio ^b^		0.7 (0.3–1.9)
Viral RNA copies/mL plasma ^b^		<50

^a^ Infection with hepatitis B or C virus. ^b^ At the time of sample collection. ^c^ ART including b-PI. ^d^ ART with no b-PI. MSM: men having sex with men; IDU: intravenous-drug user. NRTI: nucleoside analog reverse-transcriptase inhibitor; NNRTI: non-nucleoside analog reverse-transcriptase inhibitor; PI: protease inhibitor; INSTI: integrase strand transfer inhibitor.

**Table 2:** Frequency of individuals with neutralization breadth.

		Non-b-PI (*n* = 56) ^a^		b-PI (*n* = 45) ^b^	
Neutralization Category	Neutralization Score ^c^	*n*	(%)	*n*	(%)
Elite	14–18	4	7.1	1	2.2
Broad	10–13	22	39.3	4	8.9
Cross	5–9	23	41.1	28	62.2
Weak or none	<5	7	12.5	12	26.7

^a^ ART including non-boosted protease inhibitors (Non-b-PI). ^b^ ART including boosted protease inhibitors (b-PI). ^c^ A cumulative score was computed for each plasma sample as described previously [22]. Thresholds for the cumulative scores were determined to categorize plasma samples as elite, broad, cross, or weak/no neutralization.

**Table 3:** Association of different quantitative variables with neutralization score values.

Variable	Individuals ^1^	Rho	*p*-Value ^2^
Age	98	0.058	0.57
Time post-infection (years) ^3^	101	0.153	0.13
Time on ART (years) ^3^	101	−0.147	0.14
Time to ART (years) ^3^	101	0.181	0.07
CD4+/mm^3 3^	72	−0.072	0.55
CD8+/mm^3 3^	72	0.224	0.06
CD4+/CD8+ ratio ^3^	72	−0.278	**0.018**
CD4+ Nadir	100	0.000	1.00
Peak Viral Load	78	−0.069	0.55

^1^ Number of individuals with data. ^2^ Statistically significant values are in bold. ^3^ At the time of sample collection.

**Table 4:** Association of different qualitative variables with neutralization score values.

Variable		Category	Individuals	Neut. Score (Mean)	95% CI ^1^	*p*-Value ^2^
Gender		Male	89	7.8	7.1–8.5	0.17
		Female	12	6.8	4.4–9.1	
Risk group ^3^		Heterosexual	22	7.5	5.8–9.2	0.62
		MSM	68	7.7	6.9–8.5	
		IDU	4	9.3	3.6–15	
		No data	7	6.7	4.6–8.8	
Hepatitis ^4^		No	88	7.5	6.8–8.2	0.69
		Yes	3	9.0	2.9–15	
		No data	10	8.5	5.9–11	
Drug Regimen ^5^		2NRTI + 1NNRTI	53	9.1	8.1–10	**0.0001**
		2NRTI + 1 b-PI	44	6.1	5.3–7.0	
		Other	4	6.0	2.5–9.5	
b-PI		Yes	45	6.1	5.3–6.9	**<0.0001**
		No	56	8.9	8.0–9.9	
b-PI	Atazanavir + Ritonavir	Yes	24	6.1	5.3–7.0	**0.014**
		No	77	8.1	7.3–9.0	
	Lopinavir + Ritonavir	Yes	21	6.0	4.6–7.4	**0.012**
		No	80	8.1	7.3–8.8	
NNRTI	Efavirenz	Yes	46	9.1	8.1–10	**0.0001**
		No	55	6.4	5.6–7.2	
	Nevirapine	Yes	9	8.7	6.6–11	0.26
		No	92	7.6	6.8–8.3	
NRTI	Lamivudine	Yes	23	7.8	6.2–9.4	0.90
		No	78	7.6	6.8–8.4	
	Tenofovir	Yes	76	7.6	6.8–8.4	0.78
		No	25	7.9	6.3–9.4	
	Emtricitabine	Yes	75	7.6	6.8–8.4	0.92
		No	26	7.8	6.3–9.2	
	Zidovudine	Yes	22	8.1	6.4–9.7	0.63
		No	79	7.5	6.8–8.3	
	Other ^6^	Yes	5	5	1.9–8.5	0.12
		No	96	7.7	7.1–8.5	

^1^ Confidence interval. ^2^ Statistically significant values are in bold. ^3^ MSM: men having sex with men; IDU: intravenous-drug users. ^4^ Infection with hepatitis B or C virus. ^5^ NRTI: nucleoside analog reverse-transcriptase inhibitor; NNRTI: non-nucleoside analog reverse-transcriptase inhibitor; b-IP: boosted protease inhibitor (protease inhibitor + ritonavir). Other: includes 1 regimen with 3 NRTI, 2 with 1NRTI + 1 NNRTI + 1 PI, and 1 with 2 NRTI + 1 INSTI (integrase strand transfer inhibitor. ^6^ Other NRTI: includes 4 patients treated with didanosine and 1 with abacavir.

**Table 5:** Multiple linear regression model for predicting neutralization scores.

Variable	Coefficient	SE ^1^	*p*-Value ^2^	Beta
Time to ART	0.13	0.08	0.081	0.19
CD4+/CD8+ ratio	−2.0	1.2	0.084	−0.19
Efavirenz	1.2	1.1	0.269	0.16
b-PI ^3^	−2.1	1.1	**0.049**	−0.30
Constant	8.7	1.4	<0.001	

^1^ SE: standard error. ^2^ Statistically significant values are in bold, ^3^ Boosted protease inhibitor (PI + ritonavir).

## Data Availability

The data are contained within the article.
